# Anthropogenic fire reduces migratory bird abundance and diversity at a stopover site

**DOI:** 10.1038/s41598-025-27464-1

**Published:** 2025-11-18

**Authors:** Wieland Heim, Lara Hinninger, Sergei M. Smirenski, Ramona J. Heim

**Affiliations:** 1 Institute of Biology and Environmental Sciences, University of Oldenburg, Oldenburg, Germany; 2 Department of Evolutionary Biology and Environmental Studies, University of Zurich, Zurich, Switzerland; 3 International Crane Foundation, Baraboo, WI USA; 4 Institute of Landscape Ecology, University of Muenster, Muenster, Germany

**Keywords:** Biodiversity, Animal migration

## Abstract

The frequency and extent of fires are increasing worldwide. Fires impact biodiversity, including bird communities. However, our knowledge is biased towards breeding bird communities, with fire impacts on birds during migration being poorly studied. Migratory birds rely on suitable stopover sites, and any changes at such sites could negatively affect their survival and population dynamics. We investigated the effects of an anthropogenic fire on birds captured at an autumn stopover site, comparing data from one year with fire to four years thereafter without. We found significant negative effects of fire on bird abundance and diversity, though species composition remained largely unchanged. We posit that reduced habitat and food availability after fire cause these effects. We also assessed variation in stopover duration and fuel deposition rate in two species, but found no overall significant fire-related effects in either case. Management strategies should aim at limiting the extent of fires to sustain suitable stopover habitat patches.

**Supplementary Information:**

The online version contains supplementary material available at 10.1038/s41598-025-27464-1.

## Introduction

Fire is a natural evolutionary force that profoundly shapes most terrestrial ecosystems, making it a pivotal driver of biodiversity across the globe^1,2^. Disturbance through fire has the power to alter the structure, composition, and functioning of ecosystems, leading to shifts in the abundance and distribution of species^3^. The impact of fire on biodiversity can be both positive and negative, depending on specific ecosystem characteristics and fire properties^1^. This consideration is particularly crucial for biodiversity as fire regimes undergo transformations due to the climate change and other anthropogenic impacts, including land use changes^4,5^.

Birds, as indicators of ecosystem functioning, are sensitive to changes in their environment, especially to those affecting habitat structure and resource availability^6,7^. Fire can thus strongly impact bird communities by altering vegetation and other resources they require to thrive, causing immediate impacts, such as the loss of nesting sites or food sources, and longer-term effects, when habitat slowly recovers after fire^8–10^.

Fire impacts on birds have been predominantly studied within breeding habitats. Many studies reveal reduced bird abundance and species richness in the weeks or months after a fire^11,12^. Especially at high frequencies, fires have negative effects on the occurrence of many bird species^13^. Conversely, fires at low frequencies can enhance breeding bird diversity and abundance^9,14–16^.

The narrow focus on breeding birds leaves a notable gap regarding the consequences of fires on bird communities at stopovers sites during migration^17^. Stopovers are interruptions of migratory flights to minimize immediate or delayed fitness costs^18^. Migratory birds use stopover sites for example for physical recovery after endurance flights, for re-fueling of energy reserves to continue migration, or for seeking shelter during weather conditions unsuitable for migration^18^. During their annual cycle, migratory birds rely on a multitude of such sites, and changes at any of these sites can disrupt their migration and negatively affect their survival^18,19^. Fires fundamentally alter stopover site conditions for migratory birds: (1) Active fires or smoke clouds arising from fires could deter migrating birds. A study on Greater White-fronted Geese *Anser albifrons* revealed changes in migration routes due to wildfires smoke, resulting in increased flight times and potential higher mortality^20^. (2) Fire changes the stopover habitat by burning the vegetation. Fires destroy most of the herbaceous layer^10^, potentially reducing habitat e.g. for reed-dwelling bird species. Conversely, burnt areas in the southern Great Plains, USA, proved highly important stopover habitat for migratory shorebirds^21^. (3) Fire affects food availability for re-fueling. Burning grasses may impact seed-eating species because of a lack of food. On top of that, fewer insects are typically found in burnt vegetation directly after fire^22,23^, while those insects that survive might be more exposed and become more easily available as prey. Consequently, fire likely affects the quality of a stopover site, potentially leading to: (1) Site avoidance, resulting in lower bird abundance and/or diversity. Since fire effects are likely species-specific (with seed-eating grassland species likely more affected than omnivorous forest species), we expect significant changes in species composition. (2) Shortened stopover duration, due to unsuitable conditions. (3) Reduced fuel deposition rates and body mass gain, due to decreased food availability.

Evaluating the impact of fire-induced changes at stopover sites on bird diversity and abundance is essential to guide conservation efforts^21,24^. In addition to land use change, pollution and human activity, changes in fire regimes have emerged as a crucial conservation concern for migrating birds^21,25^. Both fire and fire suppression endanger 191 globally threatened bird species^26^. Future projections for fire regimes in northern Eurasia indicate a substantial rise in wildfire area and recurrence rates^27,28^, likely putting additional pressure on bird populations already suffering from other global change impacts.

Our study focuses on the impact of an anthropogenic fire at Muraviovka Park, Amur region, in the Russian Far East. This wetland ecosystem along the Amur River serves as a crucial breeding and stopover site for almost 300 bird species^29^, offering an ideal setting for investigating fire effects on migratory birds. While natural fires have historically occurred in the area, climate change and regional human activities have increased their frequency and extent^30^, leading to annual fires in the study region. Using standardized bird ringing data collected over five autumn seasons—one of which coincided with a fire event during peak migration—we aim to assess how bird communities at this important stopover site are affected by fire.

We hypothesize that:

1. Bird abundance and species richness are lower in the period immediately post-fire compared to equivalent periods in non-fire years.
2. Bird species composition differs immediately post-fire compared to equivalent periods in non-fire years.
3. Birds show decreased stopover duration, body mass gain and fuel deposition rates immediately post-fire compared to equivalent periods in non-fire years.
4. Fire effects vary among species.

## Results

We analyzed 9060 bird captures of 92 species, individually marked with numbered metal rings during our five-year survey period. We found significant negative effects of fire on bird abundance, bird species richness, and the re-fueling rate in one species. Bird species composition remained similar.

### Fire effects on bird abundance

**Fig. 1:**
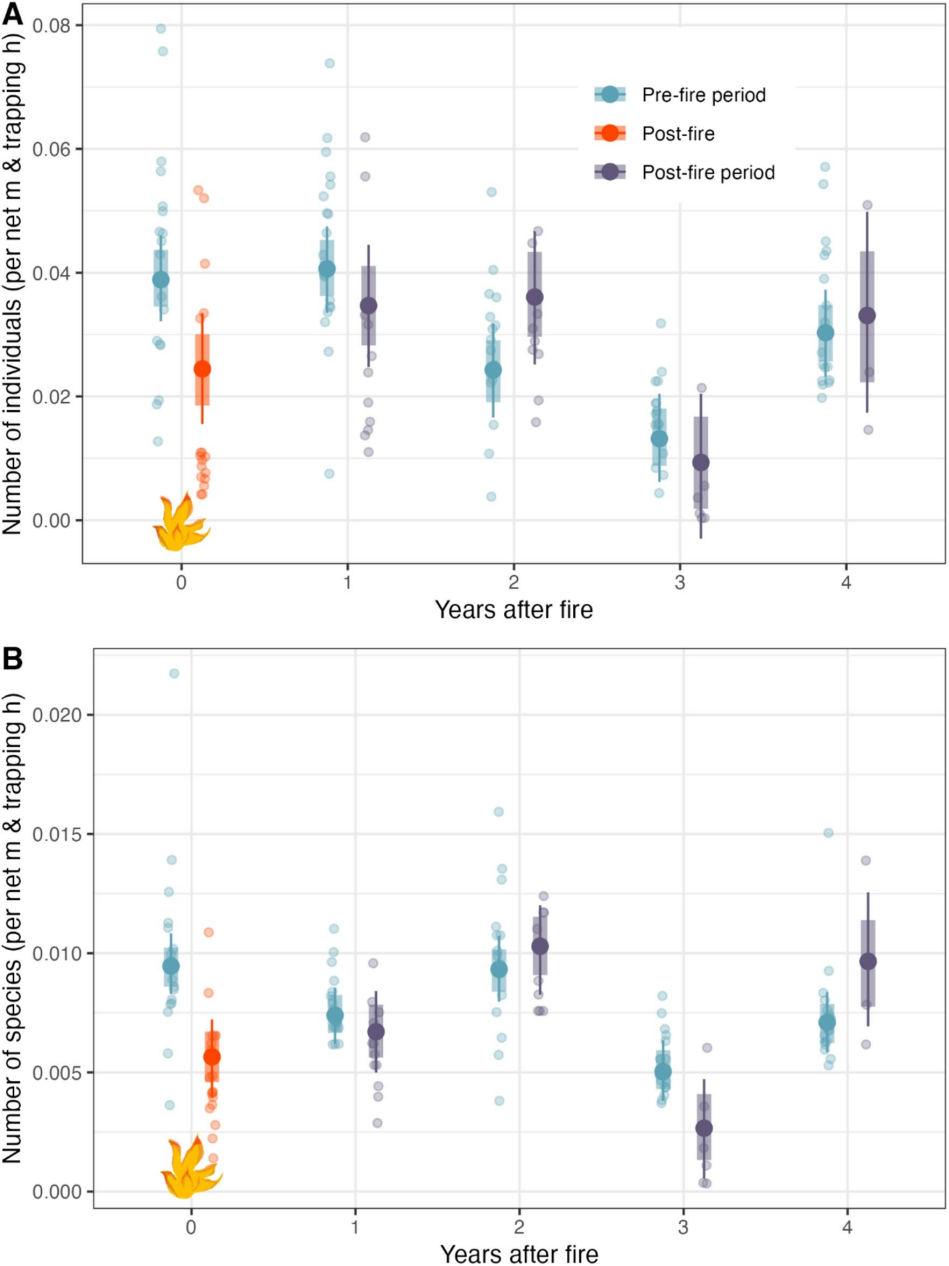
Abundance (**A**) and species richness (**B**) of birds from one fire year and the four years after, comparing pre- and post-fire periods. The predicted means are represented by large dots, with 95% credible intervals shown as thin lines and 80% credible intervals as thick lines. Each transparent dot indicates one day. The pre-fire season, or the time before the date of the fire in the fire year, is denoted by the blue color. The post-fire period is represented by orange and violet, with the orange dots in the fire year indicating the time immediately after the fire, and the violet dots serving as the control for the years without fire.

Bird abundance was slightly lower in the post-fire period compared to pre-fire conditions within the same year (80% CrI’s are not overlapping, Fig. 1, Table S1). For all other years included in the analysis, bird abundance did either not differ between the two periods (1, 3 and 4 years after fire) or were slightly increased (2 years after fire, Fig. 1, Table S1). Bird abundances differed between years.

### Fire effects on bird species richness

We found a significant negative fire impact on bird species richness—the species richness was lower in the period after fire compared to the time period before fire of the same year (95% CrI’s are not overlapping, Fig. 1, Table S2). For the third year after fire a lower species richness was also detected for the post-fire period compared to the pre-fire period and for the fourth year after fire a higher species richness was detected for the post-fire period. In these two cases, 95% CrI’s overlap, but 80% CrI’s do not.

### Fire effects on species composition

**Fig. 2:**
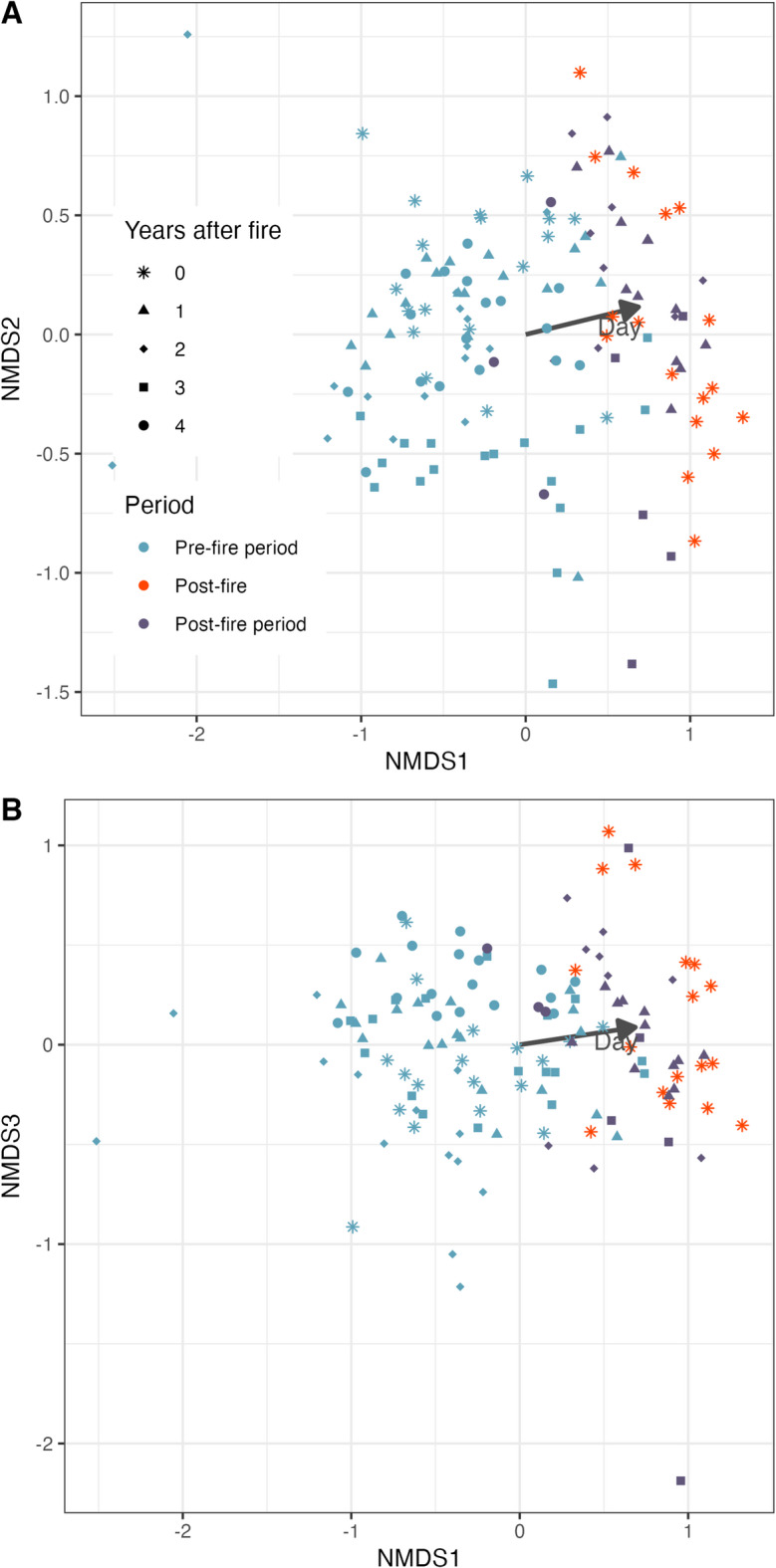
Bird species composition in years with and without fire. The non-metric multidimensional scaling (NMDS) ordination plot shows the similarities and differences between samples in a multidimensional dataset. The distances between points on the plot represent the relative dissimilarity between samples, with closer points indicating more similar samples. We overlaid the ordination with the variable “Julian day” to visualize its correlation with the NMDS dimensions. We run the NMDS with three dimensions. Dimensions 1 and 2 are shown in (**A**). Dimensions 1 and 3 are shown in (**B**). The stress value of the NMDS was 0.129. The pre-fire season, or the time before the fire in the fire year, is denoted by the blue color. The post-fire period is represented by orange and violet, with the orange dots indicating the time immediately after the fire in the fire year, and the violet dots serving as the control based on the four years after (without fire).

Fire did not affect the overall species composition (Fig. 2). The NMDS plot revealed that the datapoints mainly arranged along the Julian day gradient. There was a relatively clear separation between the pre- and post-fire periods suggesting that there is a general difference in species composition between the two periods across years (Fig. 2). The data from the post-fire period of the fire year and the other non-fire years overlapped suggesting that species composition did not differ between the year with fire and the years without (Fig. 2).

### Species-specific fire effects: abundance

**Fig. 3:**
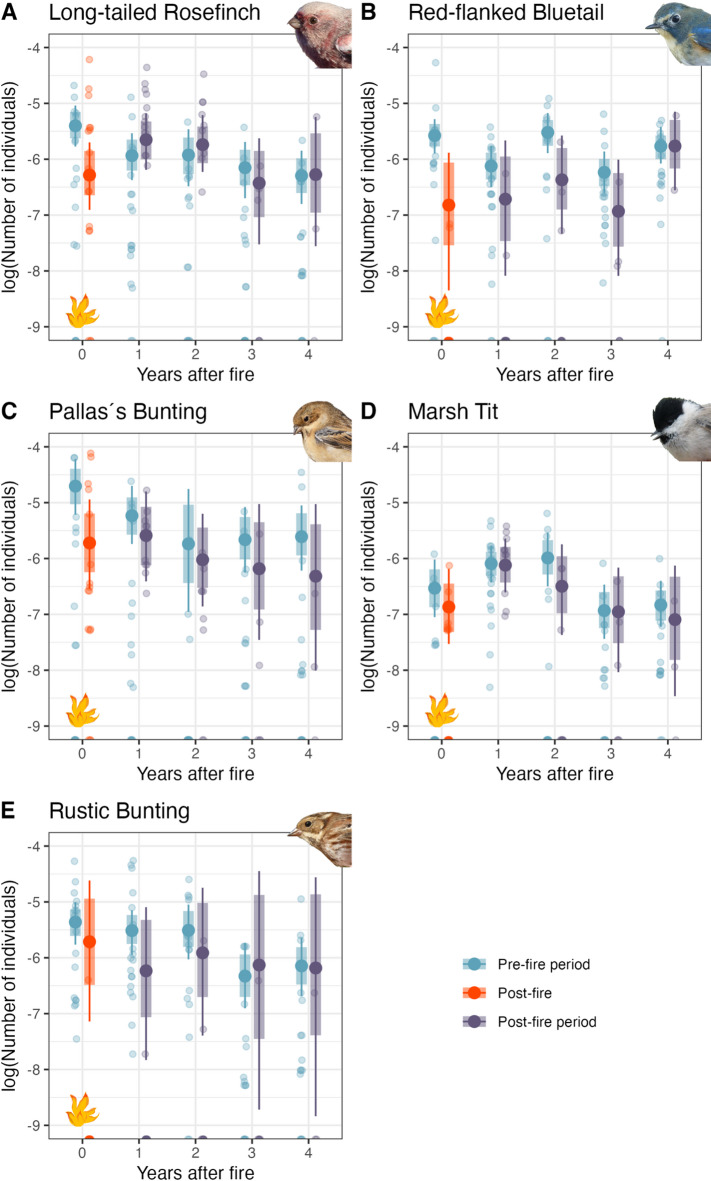
Predicted abundance (number of captured individuals corrected for net meter and trapping hours) of the five most common species across five years, comparing pre- and post-fire periods. The predicted means are represented by large dots, with 95% credible intervals shown as thin lines and 80% credible intervals as thick lines. Each transparent dot indicates one day. The pre-fire season, or the time before the fire in the fire year, is denoted by the blue color. The post-fire period is represented by orange and violet, with the orange dots indicating the time immediately after the fire in the fire year, and the violet dots serving as the control based on the four years after (without fire).

We found that fire slightly negatively impacted the number of caught individuals for three (Long-tailed Rosefinch *Carpodacus sibiricus*, Red-flanked Bluetail *Tarsiger cyanurus* and Pallas’s Bunting *Emberiza pallasi*) of the five most abundant and commonly caught species, as the 80% CrI between pre- and post-fire period of the fire year did not overlap (95% CrI’s did overlap) (Fig. 3, Tables S3–S7). For the four years after fire, the 80% CrIs for the abundance of the three species overlapped between the pre- and post-fire periods. One exception occurred two years after fire, when Red-flanked Bluetail numbers were lower post-fire but the 80% CrI did not overlap. For the other two common species (Marsh Tit *Poecile palustris* and Rustic Bunting *Emberiza rustica*) no differences between pre- and post-fire periods could be detected in any of the years (Fig. 3).

### Species-specific fire effects: stopover duration and body condition

**Fig. 4:**
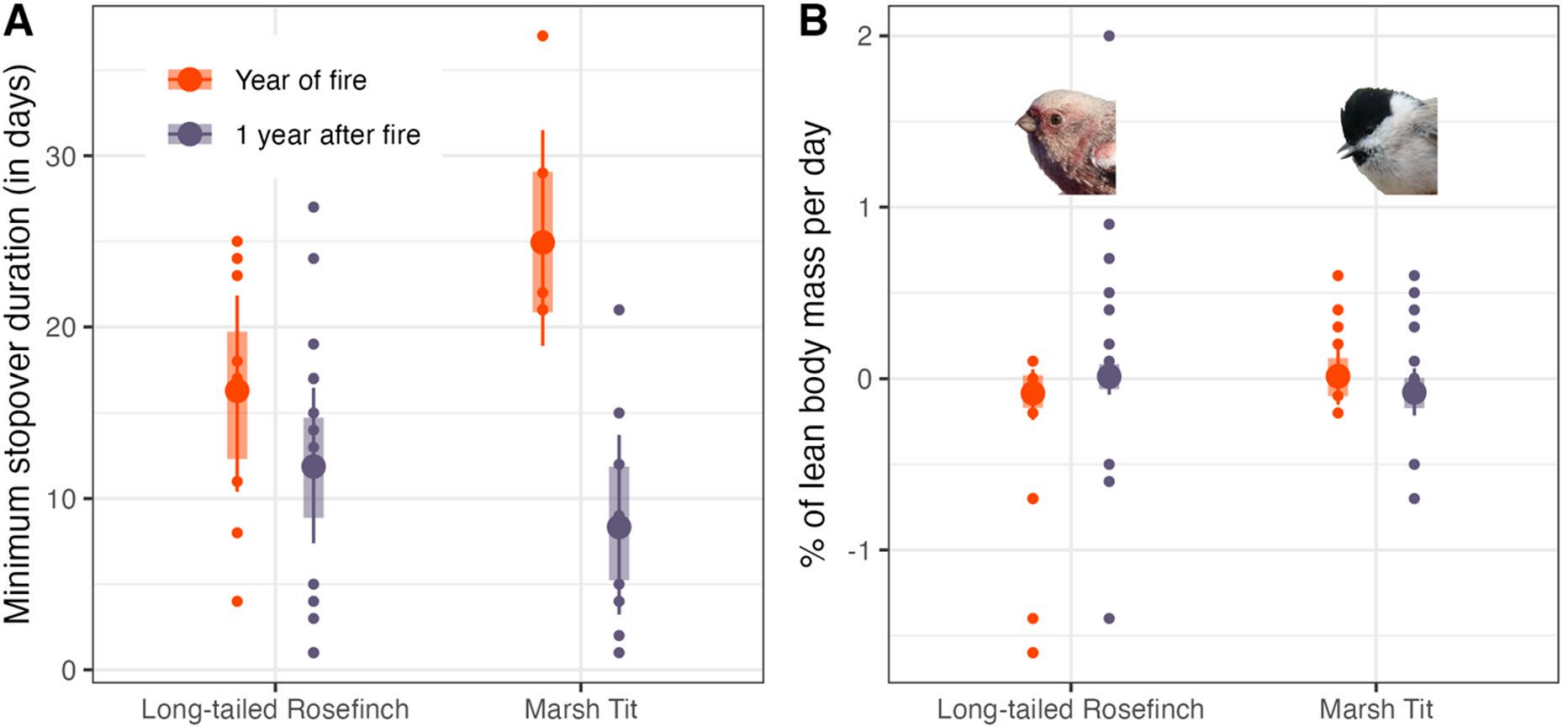
Predicted duration of stay in the study area (**A**) and predicted fuel deposition rate (in % of lean body mass per day) during the stay (**B**) of individual Long-tailed Rosefinches and Marsh Tits in the fire year (orange) and the year after (no fire, violet). The predicted means are represented by large dots, with 95% credible intervals shown as thin lines and 80% credible intervals as thick lines. Small dots represent observations for single individuals (meaning the actual measured value for either the duration of that bird’s stay in the study area or the change in its body mass during the stay).

The available number of recaptured individuals allowed to investigate minimum stopover duration and changes in body mass, fat and muscle score for two species, Long-tailed Rosefinch and Marsh Tit, in the fire year (n = 8 and 6, respectively) and the year after fire (n = 13 and 9, respectively). We found that Marsh Tits were staying significantly longer in the year of the fire compared to the following year without fire (no overlap of 95% CrI’s, Fig. 4A, Table S8), but no effect was found on stopover duration in Long-tailed Rosefinches (Table S8). We found no difference in fuel deposition rate in Marsh Tits and Long-tailed Rosefinches in the year of fire (overlap of 95% CrI with y = 0) and no differences between the years (no overlap of 95% CrI’s, Fig. 4B, Table S9). We found no significant impacts of fire on changes in fat and muscle scores in individuals of the two species during stopover (Tables S10–S11).

## Discussion

Our study revealed fire-induced reductions in the abundance and the number of species captured at a stopover site during autumn migration. However, species composition remained largely unaffected. At the species level, three of the five most common species—Long-tailed Rosefinch, Red-flanked Bluetail and Pallas´s Bunting—exhibited lower numbers after fire. Furthermore, Marsh Tits showed an increased stopover duration after fire, whereas no effect of fire was found on fuel deposition rates.

### Fire effects on bird abundance

The observed post-fire decline in bird captures contrasts sharply with the stable or increased numbers during equivalent periods in non-fire years. This pattern aligns with previous studies on breeding birds in wetland habitats, where similar reductions in abundance were documented immediately after fires in the Russian Far East and Poland^12^. Reasons for the reduced numbers of birds could include (1) deterrence of birds due to active fires and smoke clouds^31^ or (2) lack of suitable habitat needed for resting and of food needed for re-fueling during stopover^18^. For example, grasses and herbs are combusted completely through fire, thus leading to a lack of cover (as protection from severe weather conditions or predators) for species that prefer reedbeds or thickets during stopover, such as the Pallas´s Bunting at our study site^32^. The combustion of aboveground biomass through fire comprises also fruits and seeds, important food for many of the species in our study. Long-tailed Rosefinches feed on *Artemisia* seeds in autumn^33^, which completely lack after fire. Litter cover is also reduced after fire^34^, potentially contributing to lower invertebrate abundance^35^. This could limit the food availability for species feeding on insects, such as the Red-flanked Bluetail in our study^36^.

Given all the factors above that could negatively affect stopover quality, birds may opt not to stop at the site but to continue migration towards more suitable stopover areas^37^, thus leading to the reduced numbers of captured birds we have observed directly after fire. Nevertheless, the potential influence of altered mist-net visibility post-fire on capture rates, due to changes in vegetation cover and color, cannot be entirely discounted.

### Fire effects on bird species richness

The decline in bird species richness directly after fire was even stronger than the decrease in overall bird abundance at our study site (Fig. 1B). As mentioned above, the lack of specific habitat components or food sources might have prevented certain species from utilizing the stopover site. For example, not a single Siberian Accentor *Prunella montanella* was captured after the fire, whereas this species was regularly captured until the end of October in all years without fire (Table S12). Siberian Accentors prefer dense thickets during migration^38^, and might therefore have avoided the rather open, burnt landscape after the fire.

On top of that, the decline of vegetation structural heterogeneity after fire might limit the number of available niches and resources at the stopover site^32,39^. Insufficient resources after fire may intensify competition between species, which could explain the lower number of species after fire. Declines in the number of breeding bird species shortly after fire were also linked to the loss of specialized species, such as reed or wetland specialists, in other studies^12,40,41^. However, the fire happened late in the autumn season at our study site, when most reed specialists (such as reed and grasshopper warblers) had already departed^42,43^.

### Fire effects on species composition

While seasonal shifts in species composition were evident between pre- and post-fire periods, there was no clear clustering between the year with fire and the following years without fire (Fig. 2). We can infer that most species that occur usually in the post-fire period also used the stopover site directly after fire (with exceptions, see above). This suggests that while the fire reduced species richness, it may not fundamentally alter the overall species composition of the bird community. Contrasting results were obtained for species composition of breeding birds in other studies, where fire had a significant effect on the community structure in forest and tundra ecosystems^9,44,45^. This might be linked to the fact that many migratory bird species occupy different niches during breeding and non-breeding^46^. Migratory songbirds are often more specialized in breeding habitat use, but more generalist in habitat use during migration^47^, as has been documented for *Emberiza* buntings in our study area as well^32,40^. Although most species in our study likely found suitable niches within the post-fire landscape, the overall reduction in capture rate may have decreased the probability of detecting rare species, thereby influencing our estimates of species richness to a greater extent than those of species composition**.**

### Species-specific fire effects on abundance

We found a significant negative effect of fire on the number of three of the five most common bird species captured during autumn migration (Fig. 3). The lower numbers of Long-tailed Rosefinches, Red-flanked Bluetails and Pallas´s Buntings in the post-fire period of the fire year might be linked to a lack of specific resources, e.g. because seeds or insects were combusted by the fire (as discussed above).

The two species for which no fire effect was detected might be able to exploit broader niches and might therefore be able to compensate for fire-induced changes in resource availability. For example, the niche breadth of Rustic Buntings (not affected by fire) was found to be almost twice as high compared to Pallas´s Buntings (affected by fire) during autumn migration at our study site^32^.

No change in abundance after fire was also detected for Marsh Tits. Some of the Marsh Tits captured and ringed at our study site (< 30%; W. Heim, unpublished data) belong to the local, sedentary breeding population while most are dispersing individuals, that will migrate short distances. This stands in contrast to the other four species, where almost all individuals are true migrants. We argue that birds of the local population might not leave the area after fire, whereas newly arriving migrant individuals likely select other, unburned stopover sites. A possible explanation for this could be that local birds know the site much better, and can therefore find resources also under more difficult conditions after fire. On top of that, Marsh Tits are known to hoard food^48^, which they feed on during times of food scarcity (e.g. during winter, but possibly also after fire). This might offset any negative impacts of fire for this species.

Another reason for the species-specific responses to fire might be linked to migration strategy. The same stopover site might be used for different purposes by different species or individuals, e.g. for recovery or for re-fueling, resulting in different stopover durations linked to individual fitness costs^18^. While short stopovers might only serve for recovery after migratory flights, long stopovers might be needed to accumulate fuel stores for long-distance flights ahead. While burned habitats might still be suitable for short-time recovery, they might be less suitable for longer-term accumulation of fuel reserves. However, there is no obvious difference in mean stopover duration at our study site between species affected by fire, such as Pallas´s Bunting (mean stopover duration 4.7 days^49^) and Red-flanked Bluetail (mean stopover duration 3.4 days^50^), and species not affected by fire, such as Rustic Bunting (mean stopover duration 4.4 days^49^).

Most of the common species in our study are songbirds inhabiting reeds or shrubs, and are therefore likely negatively affected by fire-induced habitat changes. Other species that prefer more open habitats, such as waders, might benefit from the post-fire landscape^21^.

### Species-specific fire effects on stopover duration and fuel deposition rate

Out of the two species that we assessed, one showed a significantly longer stopover duration in the fire year, whereas no difference was found in the second species (Fig. 4A). This is contradicting our assumption that lower stopover site quality would lead to shorter stopover durations, as we expected birds to relocate to nearby more suitable areas. However, this particular species, the Marsh Tit, is only a partial migrant, as some individuals (< 30%; W. Heim, unpublished data) stay at the study site during winter, which could affect our stopover duration estimates. It is also important to highlight again that no effect was found on the abundance of this species after fire (Fig. 3D). As mentioned above, the food-hoarding behavior of this species^48^ could offset any negative fire effects.

We found no significant effects of fire on fuel deposition rates (Fig. 4B). Given that suitable, unburned habitats were nearby, individuals might have relocated to such sites to resume re-fueling^51^. However, our data was limited to two short-distance migrants, while stronger effects might be expected for long-distance migrants, which generally carry larger fuel stores and show higher fuel deposition rates at our study site^52^.

## Conclusions

Our findings highlight significant and species-specific impacts of fire on migratory birds at stopover sites. While localized fires may allow birds to find alternative nearby stopover locations, large-scale fires could significantly impair migration success by reducing habitat quality across extensive areas. This may increase the fitness costs for migratory birds, e.g. due to increased settling costs or slower re-fueling, which could ultimately lead to reduced survival rates^18^.

Given the projected changes in fire regimes globally, management strategies should focus on maintaining a diverse mosaic of habitats. By carefully managing fire extent and frequency, we can ensure a variety of suitable patches for migratory birds with different stopover habitat preferences. Future research should focus on comparing the quality of burned and unburned stopover sites within the same year, particularly examining differences in stopover durations and fuel deposition rates. Such studies will provide crucial insights for effective conservation of stopover habitats in fire-prone regions.

## Methods

### Study site and data collection

The study was conducted as part of the Amur Bird Project at Muraviovka Park, Amur region, Russia. The non-governmentally managed nature reserve is situated on the Zeya-Bureya plain, approximately 50 km southeast of Blagoveshchensk, along the Amur River. This area is an important stopover site for songbirds migrating along the East Asian Australasian flyway (EAAF)^53^. Recognized as the most species-rich migration route, this flyway hosts nearly 600 bird species, with billions of individuals undertaking annual migrations between breeding and non-breeding areas^53,54^. Currently, numerous bird species along this migration route experience concerning population declines^54^.

To capture and mark birds, around 15 mist-nets (> 150 m total length) were strategically placed in various habitats, including reedbeds, wet sedge meadows interspersed with willows, raspberry thickets, mixed forests with a rich shrub layer, and a coniferous plantation (for further details see^32,55–57^. Mist-nets were open and checked hourly between one hour before sunrise and one hour after sunset during suitable conditions, while nets were closed and no birds captured during single days with heavy rain, snowfall or strong winds. All birds were ringed with an individually numbered ring provided by the bird ringing center of Russia. Before birds were released back into the wild, we measured maximum wing length as well as body mass and estimated fat score and muscle score^58^.

On October 9, 2011, a fire took place at the study site, that burned > 5800 ha. This fire was a result of intentional arson, while most fires in the region are due to uncontrolled burning of harvested fields or garbage dumps^40^. All mist-netting sites were affected by the fire (Figure S13). No fire hit the area around the ringing station in the four following years. The fire lasted for one day and spread quickly through the area due to strong winds, burning most of the leaves, reeds and grasses as well as the litter cover while sparing large tussocks and stems of shrubs, which is typical for fires in this region^10,33^. Unburned patches of vegetation survived the fire in < 1 km distance from the study site.

All experiments were performed in accordance with the ARRIVE guidelines and relevant regulations of the Russian Federation. All experimental protocols were approved by the Federal Service for Supervision of Natural Resources (Rosprirodnadzor) and the ethics committee of the Zoological Institute, Russian Academy of Science, and no specific permissions were required according to Section 44 and Section 6 of the Federal Law of the Russian Federation No. 52. A permission to capture and ring birds at Muraviovka Park was granted by the board of directors of Muraviovka Park and the Bird Ringing Centre of Russia, Russian Academy of Science, Moscow. Our activities did not include the withdrawal of investigated species from nature.

### Data preparation and statistical analysis

**Fig. 5:**
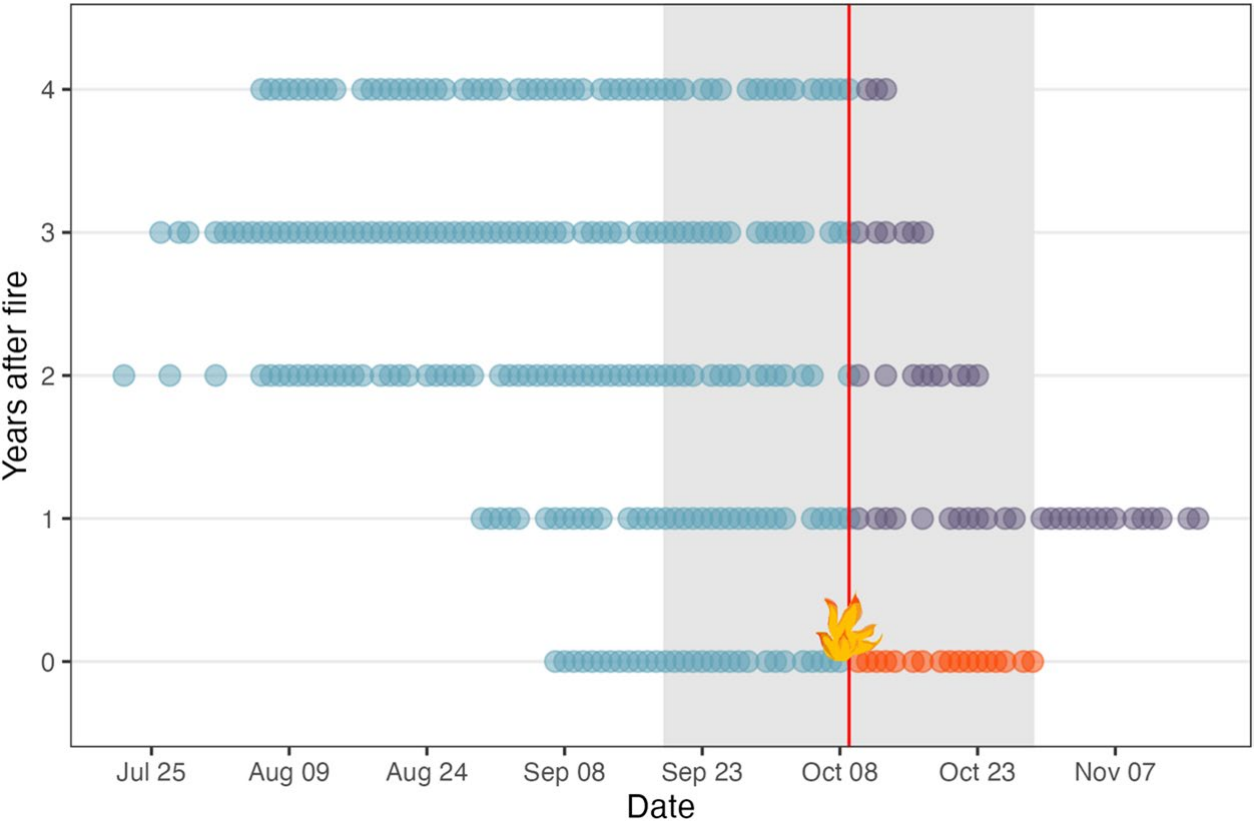
Overview on bird ringing activities at the Amur Bird Project ringing station at Muraviovka Park during the autumn seasons from 2011 to 2015. The vertical red line indicates the date of the fire event on October 9, 2011. Each point represents a day when the ringing station was operating. The blue dots correspond to the pre-fire season, which is the time before the fire occurred in 2011. The orange and violet dots indicate the post-fire period, with the orange dots in 2011 representing the time after the fire. The violet dots serve as the control without fire. The grey area shows the data extent used in this study.

For the statistical analysis, we selected data from the same timespan for all years to align with the data from the fire year (2011). The fire event occurred on Julian day 282 (9 October), and we defined the pre-fire period from day 262 to 282 (19 September to 8 October) and the post-fire period from day 283 to 302 (9 October to 29 October) for all years (Fig. 5). We corrected the daily bird abundance and species richness for trapping hours and net meters used (formulas: number of caught individuals per day / net meter / trapping hours & number of caught species per day / net meter / trapping hours).

To analyze fire impacts on bird abundance, we run a linear model with the number of caught individuals per day as dependent variable and the fire period (factor with two levels: pre-fire period, post-fire period) as well as the year (factor with five levels: 0, 1, 2, 3, 4) and the interaction of both as independent variable. In addition to that, we included Julian day as another independent variable to account for seasonal effects in our model. We run all models in R, version 4.3.0^59^ in a Bayesian framework with the brms package^60–62^. We used family = gaussian(), default brms priors and ran 2,000 iterations (warmup = 1,000) with four chains. To understand if abundances differed between periods and years, we performed post hoc comparisons with help of the emmeans package^63^ that computes the marginal means with 80 and 95% Credible Intervals (CrI’s). If these credible intervals do not overlap, we consider this difference significant. We checked the impact of song playback (which was used in some years to attract specific bird species to mist-nets) on the number of birds and species by including it in all initial models. As the variable had no significant impact in all models, we removed the variable from the final models. To analyze fire impacts on bird species richness, we used the same model structure as above, but with species richness as dependent variable.

In addition to the linear analysis, we performed an ordination to understand compositional patterns using Non-Metric Multidimensional Scaling (NMDS) with the vegan package^64^. NMDS is an ordination technique that represents the pairwise dissimilarity (in this case: Bray–Curtis) between objects in a low-dimensional space. For the input matrix, we only used data of species that occurred on at least 15 days across the previously defined timespan for all five years. In addition to that, we set the NA values in our input matrix (signifying that a species was not caught on a specific day) to 0, as they represent true absences. The initial NMDS analysis was performed using k = 2 dimensions. However, the stress value of the 2-dimensional solution was above 0.2, which indicates a poor fit of the data to the reduced 2-dimensional space. To improve the representation of the underlying dissimilarities in the data, we increased the number of dimensions to k = 3. The stress value for the 3-dimensional NMDS solution was 0.129, suggesting the 3-dimensional ordination provides a more accurate representation of the multivariate relationships in the data.

To understand fire effects on certain species we first performed the same process as described above for each of the five most abundant species using the number of caught individuals of each species as dependent variable. We included those species that were captured in all study periods (10 = 5 years * 2 fire levels) and on a total of more than 60% of the days. Five species fulfilled these specifications (ordered by the number of days in which the species occurred): Long-tailed Rosefinch (n = 603, total caught birds of that species during the study periods), Red-flanked Bluetail (n = 577), Pallas’s Bunting (n = 756), Marsh Tit (n = 322), and Rustic Bunting (n = 601). We used zero-inflated beta regression with family = zero_inflated_beta() to account for the high number of zeros indicating that a species was not caught on a certain day.

To understand differences in stopover duration or changes in body mass, fat score or muscle score, we used data of individual birds that were recaptured at least once within one year. Because it is essential that for this comparison the sampling time is the same between the years, we used the data of the years 2011 (fire) and 2012 (no fire). Minimum stopover duration was calculated as the number of days between the first and the last capture of a marked individual within the study period of one year. Note that not all individuals of all species are true migrants at our study site, as some individuals are known to overwinter in the area. We selected all species for which at least six individuals were captured first in the pre-fire period and captured a second time in the post-fire period of the same year to achieve an adequate sample size for statistical analysis. These specifications were met by two species: Long-tailed Rosefinch and Marsh Tit. With a dataset that included data for these two species we then run four linear models with the dependent variables: duration, fat change and muscle change between first capture and last recapture, with family = gaussian(). For the duration model, we included fire (yes/no), species and the interaction of both as independent variables. For the other two models we also added a random intercept for duration to control for the different time spans that the individuals had and the related possibility to gain fat score and muscle score. We furthermore run a linear model with fuel deposition rate (% of lean body mass per day) as dependent variable and fire (yes/no), species and the interaction of both as independent variables, with family = gaussian(). We calculated the fuel deposition rate using the model developed by Schaub and Jenni^64^. All our models converged successfully. Visual inspection of the posterior predictive checks suggested that the models adequately captured the patterns in the observed data.

## Supplementary Information

Below is the link to the electronic supplementary material.

Supplementary Material 1.

## Data Availability

All data are publicly available on the DARE research repository of the University of Oldenburg (DOI:10.57782/ OTDZIR).

## References

[1] He, T., Lamont, B. B. & Pausas, J. G. Fire as a key driver of Earth’s biodiversity. *Biol. Rev.* **94**, 1983–2010 (2019). 31298472 10.1111/brv.12544

[2] Pausas, J. G. & Ribeiro, E. Fire and plant diversity at the global scale. *Glob. Ecol. Biogeogr.* **26**, 889–897 (2017).

[3] Bowman, D. M. J. S. et al. Vegetation fires in the Anthropocene. *Nat Rev Earth Environ* **1**, 500–515 (2020).

[4] Kelly, L. T. et al. Fire and biodiversity in the Anthropocene. *Science* **370**, eabb0355 (2020). 33214246 10.1126/science.abb0355

[5] Kelly, L. T. et al. Understanding fire regimes for a better Anthropocene. *Annu. Rev. Environ. Resour.* **48**, 207–235 (2023).

[6] Fraixedas, S. et al. A state-of-the-art review on birds as indicators of biodiversity: Advances, challenges, and future directions. *Ecol. Indic.* **118**, 106728. 10.1016/j.ecolind.2020.106728 (2020).

[7] Morelli, F. et al. Top ten birds indicators of high environmental quality in European cities. *Ecol. Indic.* **133**, 108397 (2021).

[8] Franklin, M. J. M., Major, R. E. & Bradstock, R. A. Implications of altered fire regimes for birds of dry sclerophyll forest under climate change. *Pacific Conserv. Biol.* (2023).

[9] Heim, R. J. et al. Fire disturbance promotes biodiversity of plants, lichens and birds in the Siberian subarctic tundra. *Glob. Chang. Biol.* **28**, 1048–1062 (2022). 34706133 10.1111/gcb.15963

[10] Heim, R. J. et al. Post-burn and long-term fire effects on plants and birds in floodplain wetlands of the Russian Far East. *Biodivers. Conserv.* **28**, 1611–1628 (2019).

[11] Morales, A. M., Politi, N., Rivera, L. O., Vivanco, C. G. & Defossé, G. E. Fire and distance from unburned forest influence bird assemblages in Southern Andean Yungas of Northwest Argentina: a case study. *Fire Ecol.* **16**, 1–10 (2020).

[12] Walesiak, M., Mikusiński, G., Borowski, Z. & Żmihorski, M. Large fire initially reduces bird diversity in Poland’s largest wetland biodiversity hotspot. *Biodivers. Conserv.* **31**, 1037–1056 (2022).

[13] Reside, A. E., VanDerWal, J., Kutt, A., Watson, I. & Williams, S. Fire regime shifts affect bird species distributions. *Divers. Distrib.* **18**, 213–225 (2012).

[14] O’Reilly, L., Ogada, D., Palmer, T. M. & Keesing, F. Effects of fire on bird diversity and abundance in an East African savanna. *Afr. J. Ecol.* **44**, 165–170 (2006).

[15] Sitters, H., Di Stefano, J., Christie, F. J., Sunnucks, P. & York, A. Bird diversity increases after patchy prescribed fire: Implications from a before–after control–impact study. *Int. J. Wildland Fire* **24**, 690 (2015).

[16] Alambiaga, I., Vera, P., García, D., Rebassa, M. & Monrós, J. S. Conservation and management implications of the effects of wildfire on a threatened Eastern Iberian Reed Bunting (*Emberiza schoeniclus witherbyi*) population. *Ibis* **167**, 196–211 (2025).

[17] Schmaljohann, H., Eikenaar, C. & Sapir, N. Understanding the ecological and evolutionary function of stopover in migrating birds. *Biol. Rev.* **97**, 1231–1252 (2022). 35137518 10.1111/brv.12839

[18] Newton, I. Can conditions experienced during migration limit the population levels of birds?. *J. Ornithol.* **147**, 146–166 (2006).

[19] Kirby, J. S. et al. Key conservation issues for migratory land- and waterbird species on the world’s major flyways. *Bird. Conserv. Int.* **18**, 49–73 (2008).

[20] Overton, C. T. et al. Megafires and thick smoke portend big problems for migratory birds. *Ecology* **103**, e03552 (2022). 34622455 10.1002/ecy.3552 PMC9286671

[21] Siemann, E., Haarstad, J. & Tilman, D. Short-term and long-term effects of burning on oak savanna arthropods. *Am. Midland Naturalist.* **1**, 349–361 (1997).

[22] Parmenter, R. R., Kreutzian, M., Moore, D. I. & Lightfoot, D. C. Short-term effects of a summer wildfire on a desert grassland arthropod community in New Mexico. *Environ. Entomol.* **40**, 1051–1066 (2011). 22251717 10.1603/EN11047

[23] Carlisle, J. D. et al. Landbird migration in the American West: recent progress and future research directions. *Condor* **111**, 211–225 (2009).

[24] Haskell, L. *State of the world’s birds 2022 insights and solutions for the biodiversity crisis*. *Birdlife* (2022).

[25] Hovick, T. J., Carroll, J. M., Elmore, R. D., Davis, C. A. & Fuhlendorf, S. D. Restoring fire to grasslands is critical for migrating shorebird populations. *Ecol. Appl.* **27**, 1805–1814 (2017). 28464361 10.1002/eap.1567

[26] Pavleychik, V. Long-term dynamics of natural fires in the steppe regions (case study-of the Orenburg region). *Bullet. Orenburg State Univ.* **6**, 74–80 (2016).

[27] Shvidenko, A. Z. & Schepaschenko, D. G. Climate change and wildfires in Russia. *Contemp. Probl. Ecol.* **6**, 683–692 (2013).

[28] Heim, W. & Smirenski, S. M. The importance of Muraviovka Park, Amur province, Far East Russia, for bird species threatened at regional, national and international level based on observations between 2011 and 2016. *Forktail* **33**, 77–83 (2017).

[29] Simonov, E. A., Dahmer, T. D. & World wide fund for nature. *Amur-Heilong River Basin Reader*. (Ecosystems, 2008).

[30] Overton, C. T. et al. Megafires and thick smoke portend big problems for migratory birds. *Ecology* **103**, e03552 (2021). 34622455 10.1002/ecy.3552 PMC9286671

[31] Heim, W., Eccard, J. A. & Bairlein, F. Migration phenology determines niche use of East Asian buntings (Emberizidae) during stopover. *Curr. Zool.* **64**(6), 681–692 (2018). 30538727 10.1093/cz/zoy016 PMC6280105

[32] Clement, P. & Arkhipov, V. Long-tailed Rosefinch (Carpodacus sibiricus), version 1.0. *In Birds of the World (J. del Hoyo, A. Elliott, J. Sargatal, D. A. Christie, and E. de Juana, Editors). Cornell Lab of Ornithology, Ithaca, NY, USA.*10.2173/bow.lotros1.01 (2020).

[33] Heim, R. J. *et al.* Litter removal through fire—A key process for wetland vegetation and ecosystem dynamics. *Sci. Total Environ.* **755**, (2021). 10.1016/j.scitotenv.2020.142659 33049535

[34] Hochkirch, A. & Adorf, F. Effects of prescribed burning and wildfires on Orthoptera in Central European peat bogs. *Environ. Conserv.* **34**, 225–235 (2007).

[35] Collar, N., de Juana, E. & Christie, D. A. Red-flanked Bluetail (Tarsiger cyanurus), version 1.0. *In Birds of the World (J. del Hoyo, A. Elliott, J. Sargatal, D. A. Christie, and E. de Juana, Editors). Cornell Lab of Ornithology, Ithaca, NY, USA.*10.2173/bow.refblu.01 (2020).

[36] Ktitorov, P., Tsvey, A. & Mukhin, A. The good and the bad stopover: Behaviours of migrant reed warblers at two contrasting sites. *Behav Ecol Sociobiol* **64**, 1135–1143 (2010).

[37] Hatchwell, B. Siberian Accentor (Prunella montanella), version 1.0. *In Birds of the World (J. del Hoyo, A. Elliott, J. Sargatal, D. A. Christie, and E. de Juana, Editors). Cornell Lab of Ornithology, Ithaca, NY, USA*10.2173/bow.sibacc.01 (2020).

[38] Kober, K. & Bairlein, F. Habitat Choice and niche characteristics under poor food conditions. A study on migratory nearctic shorebirds in the intertidal flats of Brazil. *Ardea* **97**, 31–42 (2009).

[39] Heim, W. et al. Anthropogenic fire patterns affect niche breadth and niche overlap in sympatric songbird species. *Sci. Total Environ.* **833**, 155160 (2022). 35417726 10.1016/j.scitotenv.2022.155160

[40] Isacch, J. P., Holz, S., Ricci, L. & Martínez, M. M. Post-fire vegetation change and bird use of a salt marsh in coastal Argentina. *Wetlands* **24**, 235–243 (2004).

[41] Bozó, L. et al. Migration timing of Pallas’s Grasshopper–Warbler *Locustella certhiola* and *Lanceolated Warbler* L. lanceolata at a stopover site in the Russian Far East. *Ornithol. Sci.* **18**(2), 177–181 (2019).

[42] Bozó, L., Heim, W. & Csörgő, T. Habitat use by Siberian warbler species at a stopover site in Far Eastern Russia. *Ringing Migr.* **33**, 31–35 (2018).

[43] Schieck, J. & Song, S. J. Changes in bird communities throughout succession following fire and harvest in boreal forests of western North America: literature review and meta-analyses. *Can. J. For. Res.* **36**, 1299–1318 (2006).

[44] Fontaine, J. B., Donato, D. C., Robinson, W. D., Law, B. E. & Kauffman, J. B. Bird communities following high-severity fire: response to single and repeat fires in a mixed-evergreen forest, Oregon, USA. *For. Ecol. Manage* **257**, 1496–1504 (2009).

[45] Ponti, R., Arcones, A., Ferrer, X. & Vieites, D. R. Seasonal climatic niches diverge in migratory birds. *Ibis* **162**, 318–330 (2020).

[46] Petit, D. R. Habitat use by landbirds along Nearctic-Neotropical migration routes: Implications for conservation of stopover habitats. *Stud. Avian Biol.* **20**, 15–33 (2000).

[47] Cowie, R. J., Krebs, J. R. & Sherry, D. F. Food storing by marsh tits. *Anim. Behav.* **29**, 1252–1259 (1981).

[48] Collet, L. & Heim, W. Differences in stopover duration and body mass change among Emberiza buntings during autumn migration in the Russian Far East. *J. Ornithol.* **163**(3), 779–789 (2022).

[49] Bozó, L., Csörgő, T. & Heim, W. Stopover duration and body mass change of two Siberian songbird species at a refuelling site in the Russian Far East. *Ornithol. Sci.* **19**, 159–166 (2020).

[50] Baker, A. J. et al. Rapid population decline in red knots: fitness consequences of decreased refuelling rates and late arrival in Delaware Bay. *Proc. R Soc. Lond. B Biol. Sci.* **271**, 875–882 (2004). 10.1098/rspb.2003.2663 PMC1691665 15255108

[51] Sander, M. M., Heim, W. & Schmaljohann, H. Seasonal and diurnal increases in energy stores in migratory warblers at an autumn stopover site along the Asian-Australasian flyway. *J. Ornithol.* **161**(1), 73–87 (2020).

[52] Yong, D. L. et al. Migratory songbirds in the East Asian-Australasian Flyway: a review from a conservation perspective. *Bird Conserv Int* **25**, 1–37 (2015).

[53] Yong, D. et al. The state of migratory landbirds in the East Asian Flyway: distributions, threats, and conservation needs. *Front. Ecol. Evol.* **9**, 613172 (2021).

[54] Wobker, J., Heim, W. & Schmaljohann, H. Sex, age, molt strategy, and migration distance explain the phenology of songbirds at a stopover along the East Asian flyway. *Behav. Ecol. Sociobiol.* **75**, 25 (2021).

[55] Sander, M. M., Heim, W. & Schmaljohann, H. Seasonal and diurnal increases in energy stores in migratory warblers at an autumn stopover site along the Asian-Australasian flyway. *J. Ornithol.* **161**, 73–87 (2020).

[56] Heim, W., Smirenski, S. M., Siegmund, A. & Eidam, F. Results of an autumnal bird ringing project at Muraviovka Park (Amur Region) in 2011. *Avian Ecol. Behav.* **21**, 27–40 (2012).

[57] Eck, S. *et al. Measuring Birds*. (Christ Media Natur, 2011).

[58] R Core Team. R: A Language and Environment for Statistical Computing. Available online at https://www.R-project.org/ (2023).

[59] Bürkner, P.-C. brms: An R package for Bayesian multilevel models using Stan. *J. Stat. Softw.* **80**, 1–28 (2017).

[60] Bürkner, P.-C. Advanced Bayesian multilevel modeling with the R package brms. *R J* **10**, 395–411 (2018).

[61] Bürkner, P.-C. Bayesian Item response modeling in R with brms and Stan. *J. Stat. Softw.* **100**, 1–54 (2021).

[62] Lenth, R. V. *emmeans: Estimated Marginal Means, aka Least-Squares Means*. Available online at https://CRAN.R-project.org/package=emmeans (2023).

[63] Oksanen, J. *et al.* vegan: *Community Ecology Package*. Available online at https://CRAN.R-project.org/package=vegan (2022).

[64] Schaub, M. & Jenni, L. Fuel deposition of three passerine bird species along the migration route. *Oecologia* **122**, 306–317 (2000). 28308281 10.1007/s004420050036

