# Supplementary material for: Anthropogenic fire reduces migratory bird abundance and diversity at a stopover site
Source: Sci Rep. 2025 Nov 18;15:40346. doi: 10.1038/s41598-025-27464-1 (PMC12627704; doi:10.1038/s41598-025-27464-1)
Supplement: Supplementary file 1 — Supplementary Material 1. [file 41598_2025_27464_MOESM1_ESM.pdf]

Supplementary Material

Anthropogenic fire reduces migratory bird abundance and diversity at a stopover site

Wieland Heim[1+*], Lara Hinninger[2+], Sergei M. Smirenski[3], Ramona J. Heim[2,4]

[1] Institute of Biology and Environmental Sciences, University of Oldenburg, Oldenburg, Germany

[2] Department of Evolutionary Biology and Environmental Studies, University of Zurich, Zurich, Switzerland

[3] International Crane Foundation, Baraboo, Wisconsin, USA

[4] Institute of Landscape Ecology, University of Muenster, Muenster, Germany

+ equal contribution

[*] corresponding author: wieland.heim@uni-oldenburg.de

Table S1: Estimates of the model explaining bird abundance across five years for two time periods before and after the date of the fire in the fire year (fire year = Year 0).

	**Estimate**	**Est.Error**	**Q2.5**	**Q97.5**
**Intercept**	0.165	0.058	0.047	0.283
**fire_periodPostMfireperiod**	-0.014	0.006	-0.028	-0.002
**Year1**	0.002	0.004	-0.007	0.010
**Year2**	-0.014	0.005	-0.023	-0.005
**Year3**	-0.026	0.005	-0.035	-0.017
**Year4**	-0.009	0.005	-0.017	0.000
**Day**	-0.000	0.000	-0.001	-0.000
**fire_periodPostMfireperiod:Year1**	0.009	0.007	-0.005	0.022
**fire_periodPostMfireperiod:Year2**	0.026	0.007	0.011	0.041
**fire_periodPostMfireperiod:Year3**	0.010	0.008	-0.006	0.027
**fire_periodPostMfireperiod:Year4**	0.017	0.010	-0.003	0.037

Table S2: Estimates of the model explaining bird species richness across five years for two time periods before and after the date of the fire in the fire year (fire year = Year 0).

	Estimate	Est.Error	Q2.5	Q97.5
**Intercept**	0.023	0.010	0.004	0.043
**fire_periodPostMfireperiod**	-0.004	0.001	-0.006	-0.002
**Year1**	-0.002	0.001	-0.004	-0.000
**Year2**	-0.000	0.001	-0.002	0.002
**Year3**	-0.004	0.001	-0.006	-0.003
**Year4**	-0.002	0.001	-0.004	-0.001
**Day**	-0.000	0.000	-0.000	0.000
**fire_periodPostMfireperiod:Year1**	0.003	0.001	0.001	0.006
**fire_periodPostMfireperiod:Year2**	0.005	0.001	0.002	0.007
**fire_periodPostMfireperiod:Year3**	0.001	0.001	-0.001	0.004
**fire_periodPostMfireperiod:Year4**	0.006	0.002	0.003	0.010

Table S3: Estimates of the model explaining Long-tailed Rosefinch abundance across five years for two time periods before and after the date of the fire in the fire year (fire year = Year 0).

	Estimate	Est.Error	Q2.5	Q97.5
**Intercept**	-15.566	3.770	-23.132	-8.170
**fire_periodPostMfireperiod**	-0.885	0.412	-1.683	-0.074
**Year1**	-0.537	0.254	-1.048	-0.050
**Year2**	-0.529	0.296	-1.133	0.051
**Year3**	-0.755	0.296	-1.358	-0.185
**Year4**	-0.905	0.287	-1.482	-0.357
**Day**	0.036	0.014	0.009	0.064
**fire_periodPostMfireperiod:Year1**	1.174	0.342	0.526	1.846
**fire_periodPostMfireperiod:Year2**	1.085	0.391	0.311	1.884
**fire_periodPostMfireperiod:Year3**	0.566	0.606	-0.708	1.643
**fire_periodPostMfireperiod:Year4**	0.843	0.702	-0.712	2.049

Table S4: Estimates of the model explaining Red-flanked Bluetail abundance across five years for two time periods before and after the date of the fire in the fire year (fire year = Year 0).

	Estimate	Est.Error	Q2.5	Q97.5
**Intercept**	-8.597	3.008	-14.559	-2.431
**fire_periodPostMfireperiod**	-1.350	0.694	-3.010	-0.290
**Year1**	-0.544	0.220	-0.983	-0.117
**Year2**	0.061	0.219	-0.389	0.480
**Year3**	-0.655	0.230	-1.116	-0.214
**Year4**	-0.184	0.210	-0.592	0.231
**Day**	0.011	0.011	-0.012	0.033
**fire_periodPostMfireperiod:Year1**	0.639	0.974	-1.291	2.543
**fire_periodPostMfireperiod:Year2**	0.459	0.836	-1.061	2.223
**fire_periodPostMfireperiod:Year3**	0.584	0.865	-1.026	2.366
**fire_periodPostMfireperiod:Year4**	1.317	0.783	-0.061	3.040

Table S5: Estimates of the model explaining Pallas´s Bunting abundance across five years for two time periods before and after the date of the fire in the fire year (fire year = Year 0).

	Estimate	Est.Error	Q2.5	Q97.5
**Intercept**	-13.554	5.120	-23.684	-3.606
**fire_periodPostMfireperiod**	-1.012	0.555	-2.089	0.067
**Year1**	-0.532	0.322	-1.132	0.113
**Year2**	-1.076	0.618	-2.445	-0.004
**Year3**	-0.957	0.360	-1.666	-0.251
**Year4**	-0.907	0.358	-1.602	-0.202
**Day**	0.032	0.019	-0.005	0.069
**fire_periodPostMfireperiod:Year1**	0.660	0.456	-0.276	1.507
**fire_periodPostMfireperiod:Year2**	0.765	0.772	-0.707	2.383
**fire_periodPostMfireperiod:Year3**	0.463	0.712	-1.014	1.769
**fire_periodPostMfireperiod:Year4**	0.242	0.907	-1.705	1.865

Table S6: Estimates of the model explaining Marsh Tit abundance across five years for two time periods before and after the date of the fire in the fire year (fire year = Year 0).

	Estimate	Est.Error	Q2.5	Q97.5
**Intercept**	-9.959	3.191	-16.158	-3.604
**fire_periodPostMfireperiod**	-0.329	0.455	-1.239	0.560
**Year1**	0.439	0.285	-0.100	1.008
**Year2**	0.519	0.355	-0.205	1.199
**Year3**	-0.402	0.349	-1.083	0.290
**Year4**	-0.292	0.320	-0.887	0.377
**Day**	0.012	0.012	-0.011	0.035
**fire_periodPostMfireperiod:Year1**	0.310	0.425	-0.554	1.136
**fire_periodPostMfireperiod:Year2**	-0.193	0.593	-1.389	0.958
**fire_periodPostMfireperiod:Year3**	0.265	0.654	-1.050	1.481
**fire_periodPostMfireperiod:Year4**	-0.040	0.789	-1.739	1.341

Table S7: Estimates of the model explaining Rustic Bunting abundance across five years for two time periods before and after the date of the fire in the fire year (fire year = Year 0).

	Estimate	Est.Error	Q2.5	Q97.5
**Intercept**	-2.893	3.630	-10.011	4.214
**fire_periodPostMfireperiod**	-0.412	0.710	-2.073	0.740
**Year1**	-0.152	0.230	-0.613	0.306
**Year2**	-0.149	0.259	-0.667	0.353
**Year3**	-0.975	0.314	-1.611	-0.368
**Year4**	-0.800	0.298	-1.407	-0.250
**Day**	-0.009	0.013	-0.035	0.017
**fire_periodPostMfireperiod:Year1**	-0.377	1.019	-2.426	1.609
**fire_periodPostMfireperiod:Year2**	-0.098	1.030	-2.269	1.912
**fire_periodPostMfireperiod:Year3**	0.466	1.300	-2.478	2.870
**fire_periodPostMfireperiod:Year4**	0.157	1.385	-3.002	2.623

Table S2: Estimates of the model explaining stopover duration of individual Long-tailed Rosefinches and Marsh Tits in the fire year and the year after fire.

	Estimate	Est.Error	Q2.5	Q97.5
**Intercept**	11.821	2.318	7.260	16.340
**fireyes**	4.515	3.667	-2.422	12.018
**Speciesmarshtit**	-3.381	3.594	-10.458	3.841
**fireyes:Speciesmarshtit**	11.998	5.685	0.764	23.118

Table S3: Estimates of the model explaining fuel deposition rates of individual Long-tailed Rosefinches and Marsh Tits in the fire year and the year after fire.

	Estimate	Est.Error	Q2.5	Q97.5
**Intercept**	-0.084	0.074	-0.233	0.064
**fireno**	0.097	0.095	-0.092	0.286
**Speciesmarshtit**	0.098	0.113	-0.132	0.314
**fireno:Speciesmarshtit**	-0.190	0.146	-0.466	0.105

Table S4: Estimates of the model explaining change in fat score of individual Long-tailed Rosefinches and Marsh Tits in the fire year and the year after fire.

	Estimate	Est.Error	Q2.5	Q97.5
**Intercept**	0.659	0.420	-0.171	1.469
**fireno**	-0.547	0.476	-1.517	0.379
**Speciesmarshtit**	0.884	0.678	-0.467	2.201
**fireno:Speciesmarshtit**	-0.359	0.760	-1.879	1.116

Table S5: Estimates of the model explaining change in muscle score of individual Long-tailed Rosefinches and Marsh Tits in the fire year and the year after fire.

	Estimate	Est.Error	Q2.5	Q97.5
**Intercept**	-0.353	0.158	-0.670	-0.044
**fireno**	0.287	0.174	-0.080	0.610
**Speciesmarshtit**	0.327	0.255	-0.184	0.826
**fireno:Speciesmarshtit**	-0.346	0.276	-0.880	0.213

Table S6: Number of birds caught per species, year and period as well as total number per species. Rows highlighted in bold are the species selected for the analysis of species specific fire effects.

	**2011**		**2012**		**2013**		**2014**		**2015**		
**Species**	**1**	**2 (fire)**	**1**	**2**	**1**	**2**	**1**	**2**	**1**	**2**	**Total**
Long-tailed Tit	136	35	349	121	19	90	14	1	381	36	1182
**Pallas´s Reed Bunting**	**199**	**100**	**168**	**114**	**7**	**15**	**51**	**10**	**89**	**3**	**756**
Little Bunting	181	4	251	3	48	0	54	0	93	2	636
**Long-tailed Rosefinch**	**101**	**82**	**82**	**152**	**24**	**55**	**43**	**11**	**41**	**11**	**602**
**Rustic Bunting**	**148**	**5**	**240**	**3**	**75**	**5**	**52**	**3**	**68**	**2**	**601**
**Red-flanked Bluetail**	**116**	**2**	**105**	**3**	**80**	**7**	**96**	**5**	**138**	**25**	**577**
Pallas´s Leaf Warbler	32	1	142	1	21	0	93	0	143	4	437
Dusky Warbler	58	1	96	0	76	0	80	0	90	7	408
Black-faced Bunting	29	0	229	0	54	2	46	0	34	0	394
Yellow-browed Warbler	44	0	87	0	20	1	54	0	175	3	384
**Marsh Tit**	**17**	**11**	**119**	**70**	**19**	**7**	**31**	**6**	**37**	**5**	**322**
Eurasian Tree Sparrow	49	71	53	67	1	16	15	4	11	17	304
Siberian Accentor	31	0	58	22	33	7	63	3	49	17	283
Siberian Rubythroat	5	0	81	0	22	0	55	0	16	0	179
Northern Great Tit	9	20	25	17	5	13	25	4	33	6	157
Brambling	11	1	104	0	22	3	1	0	13	0	155
Naumann´s Thrush	28	14	28	2	9	1	7	0	50	13	152
Olive-backed Pipit	10	0	54	0	13	0	24	0	48	1	150
Eurasian Nuthatch	1	0	54	22	8	11	6	0	17	1	120
Azure-winged Magpie	4	5	57	15	0	0	5	12	2	1	101
Elegant Bunting	9	1	16	0	14	0	3	0	35	1	79
Azure Tit	11	7	8	12	0	2	12	3	18	2	75
Common Redpoll	0	0	0	21	8	40	0	0	0	0	69
Dusky Thrush	15	0	14	1	3	1	9	0	18	4	65
Willow Tit	0	0	14	2	20	7	3	0	13	3	62
Taiga Flycatcher	12	0	16	0	3	0	2	0	17	5	55
Black-browed Reed Warbler	7	0	21	0	2	0	8	0	3	0	41
Grey-backed Thrush	5	0	8	0	10	0	7	0	11	0	41
Common Rosefinch	6	0	17	0	2	0	0	0	10	2	37
Bluethroat	1	0	12	0	4	0	13	0	2	3	35
Pine Bunting	7	0	9	0	2	9	1	0	6	0	34
Japanese Reed Bunting	2	0	28	0	0	0	1	0	1	0	32
Buff-bellied Pipit	2	1	10	2	0	0	6	0	10	0	31
Pallas´s Rosefinch	0	5	0	15	0	6	0	0	0	4	30
Lanceolated Warbler	2	0	17	0	3	0	4	0	3	0	29
Common Snipe	11	0	15	0	0	0	1	0	1	0	28
Yellow-browed Bunting	5	0	7	1	3	1	8	0	3	0	28
Eastern Great Tit	0	6	6	0	4	2	5	0	4	0	27
Radde´s Warbler	3	0	6	0	11	0	1	0	5	0	26

White-backed Woodpecker	0	2	3	4	5	6	2	1	0	3	26
Eurasian Treecreeper	0	0	3	6	4	2	3	0	4	3	25
Coal Tit	1	0	10	2	1	1	7	0	0	2	24
Eurasian Jay	1	1	1	0	3	3	4	2	5	0	20
Goldcrest	0	1	11	1	0	0	1	0	2	2	18
Lesser Spotted Woodpecker	0	1	3	4	0	0	3	0	5	2	18
Mugimaki Flycatcher	4	0	0	0	10	0	2	0	2	0	18
Grey-headed Woodpecker	0	2	1	3	2	3	2	1	0	3	17
Chestnut Bunting	7	0	7	0	1	0	1	0	0	0	16
Daurian Redstart	4	0	4	0	1	0	3	0	1	0	13
Hawfinch	0	0	0	6	0	4	2	0	0	0	12
Eurasian Bullfinch	0	0	0	11	0	0	0	0	0	0	11
Bohemian Waxwing	0	0	0	1	0	7	0	0	0	0	8
Northern Long-eared Owl	0	1	3	0	0	1	1	1	1	0	8
Swinhoe´s Robin	0	0	2	0	1	0	1	0	4	0	8
Common Magpie	0	2	0	1	0	1	1	0	1	0	6
Eurasian Siskin	0	0	5	0	0	1	0	0	0	0	6
Northern x Eastern Great Tit	0	1	1	0	1	2	1	0	0	0	6
Oriental Turtle Dove	0	0	0	0	1	0	2	1	2	0	6
Eastern Water Rail	1	0	3	0	0	0	1	0	0	0	5
Great Spotted Woodpecker	0	0	0	1	0	3	1	0	0	0	5
Winter Wren	0	0	0	0	0	1	2	0	2	0	5
Yellow-breasted Bunting	1	0	2	0	0	0	0	0	2	0	5
Common Reed Bunting	0	0	4	0	0	0	0	0	0	0	4
Eurasian Sparrowhawk	0	0	0	0	0	0	2	0	2	0	4
Grey-capped Pygmy Woodpecker	0	0	0	0	1	1	0	0	1	1	4
Oriental Greenfinch	1	0	3	0	0	0	0	0	0	0	4
Pale Thrush	0	0	0	0	0	0	2	0	2	0	4
Pin-tailed Snipe	0	0	2	0	0	0	2	0	0	0	4
Siberian Stonechat	0	0	2	0	0	0	0	0	1	0	3
Arctic Redpoll	0	0	0	2	0	0	0	0	0	0	2
Eyebrowed Thrush	0	0	0	0	0	0	0	0	2	0	2
Northern Goshawk	0	0	0	0	2	0	0	0	0	0	2
Red-throated Pipit	0	0	0	0	0	0	1	0	1	0	2
Thick-billed Warbler	0	0	1	0	1	0	0	0	0	0	2
Tristram´s Bunting	1	0	1	0	0	0	0	0	0	0	2
Two-barred Warbler	0	0	0	0	1	0	0	0	1	0	2
Brown Shrike	0	0	1	0	0	0	0	0	0	0	1
Chestnut-eared Bunting	0	0	1	0	0	0	0	0	0	0	1
Chinese Grey Shrike	0	1	0	0	0	0	0	0	0	0	1
Common Crossbill	0	0	0	0	0	1	0	0	0	0	1
Common Kingfisher	0	0	0	0	0	0	1	0	0	0	1
Eurasian Kestrel	0	0	0	0	0	1	0	0	0	0	1
Eurasian Teal	0	0	0	0	0	0	0	0	1	0	1

Great Grey Shrike	1	0	0	0	0	0	0	0	0	0	1
Grey Nightjar	0	0	0	0	1	0	0	0	0	0	1
Japanese Waxwing	0	0	0	0	0	0	0	0	1	0	1
Northern Hawk Owl	0	0	0	0	0	0	0	0	1	0	1
Pallas´s Grasshopper Warbler	0	0	1	0	0	0	0	0	0	0	1
Pine Grosbeak	0	0	0	0	0	1	0	0	0	0	1
Short-eared Owl	0	0	1	0	0	0	0	0	0	0	1
Siberian Bush Warbler	1	0	0	0	0	0	0	0	0	0	1
Yellow-browed warbler	1	0	0	0	0	0	0	0	0	0	1
Brambling	0	0	1	0	0	0	0	0	0	0	1

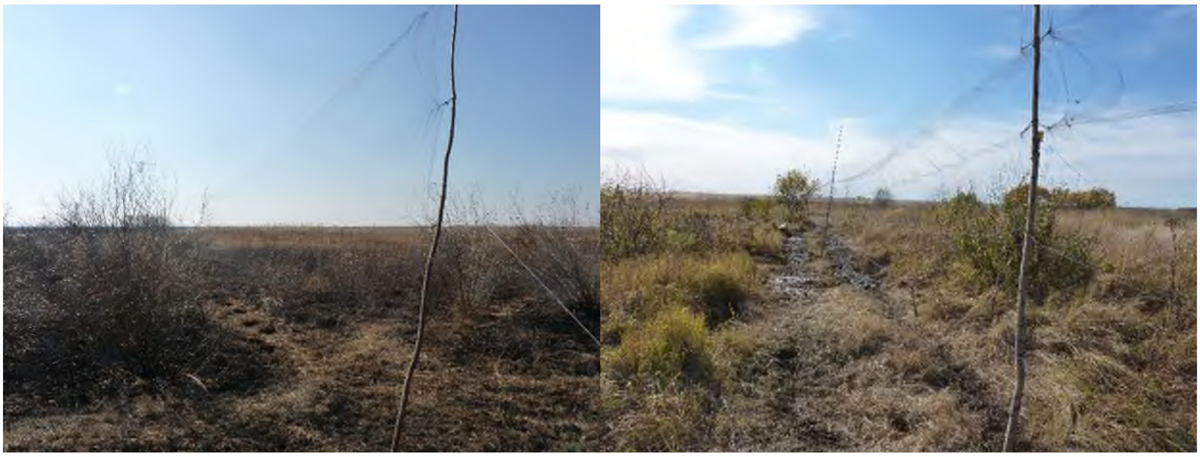

Figure S13: Mist-netting sites in the wetlands of Muraviovka Park, Russia, directly after fire (left) and in the year after fire (right). Note the lack of leaves on the shrubs and the reduction of the grass and litter cover in the fire year. Note also that soil and shrubs are black after fire but brown or yellowish in the year without fire. Photos by Wieland Heim.
